# Inclination to pursue Veterans Health Administration for primary care practice: survey of medical residents

**DOI:** 10.3389/frhs.2024.1394072

**Published:** 2024-07-18

**Authors:** Nora B. Henrikson, Megan Moldestad, Charles Maynard, Peter J. Kaboli, Ashok Reddy, Seppo T. Rinne, Karen M. Sanders, Ryan A. Sterling, Edwin S. Wong

**Affiliations:** ^1^ Kaiser Permanente Washington Health Research Institute, Kaiser Permanente Washington, Seattle, WA, United States; ^2^ Center for Veteran-Centered and Value-Driven Care, VA Puget Sound Health Care System, Seattle, WA, United States; ^3^ Department of Health Systems and Population Health, University of Washington, Seattle, WA, United States; ^4^ Comprehensive Access and Delivery Research and Evaluation Center, Iowa City VA Healthcare System, Iowa City, IA, United States; ^5^ Department of Internal Medicine, Carver College of Medicine, University of Iowa, Iowa City, IA, United States; ^6^ Division of General Internal Medicine, Department of Medicine, University of Washington, Seattle, WA, United States; ^7^ Center for Healthcare Organization and Implementation Research, VA Bedford Healthcare System, Bedford, MA, United States; ^8^ Office of Academic Affiliations, Veterans Health Administration, Washington, DC, United States

**Keywords:** workforce, survey, veteran, primary care, resident

## Abstract

**Introduction:**

Health systems like the Veterans Health Administration (VA) face challenges in recruiting and retaining a primary care physician workforce. This cross-sectional study of recent or current VA medical residents sought to identify determinants of intent to pursue primary care practice in VA after residency training.

**Methods:**

Residents were identified from administrative data between 2020 and 2021 and recruited via an emailed self-administered survey. Multivariable logistic regression, accounting for survey non-response, was applied to examine the association between intent to pursue VA practice and two sets of measures: VA training experiences and individual preferences for work conditions.

**Results:**

Of 268 responses received, 141 (56%) of the sample reported inclination to consider VA employment post-residency. Experiences with training in VA were rated more positively in the VA-inclined group compared to the not-inclined group. In the multivariable model, intent to practice primary care was the strongest predictor (OR 4.04, *p* < 0001). Preceptors' modeling of work-life balance (OR 3.23, *p* = 0.009) and perceptions of quality of clinical staff and services (OR 2.64, *p* = 0.004), ability to get patients the care they need (OR 2.51, *p* = 0.017), and quality of patient care (OR 2.30, *p* = 0.075) were independent predictors of being in the VA inclined group.

**Conclusion:**

Overall, we found that intent to practice primary care and the quality of VA training experiences are important determinants of inclination to consider VA for employment. These results provide an important perspective relevant to medical education, the hiring and retention of the United States (U.S). primary care workforce.

## Introduction

Primary care physician workforce shortages are prevalent in the United States (1, 2). Physician turnover and shortages can negatively impact patient care (3, 4). Initial exposure to an organization strongly influences the desire to stay or leave an organization (5).

The Veterans Health Administration (VA) is the largest provider of health professions training in the United States (U.S.). During the 2020–2021 academic year, approximately 113,000 health profession trainees participated in training programs at 150 Veterans Affairs Medical Centers (VAMCs) (6). Within VA, greater trainee satisfaction increases the likelihood of considering VA for employment post residency (7, 8). However, the factors influencing choice of VA for primary care practice after residency, particularly in people who have been exposed to training at VA, are not well understood. The objective of this cross-sectional survey study was to identify determinants of inclination to pursue VA for primary care practice. Our analyses focus on physician residents rotating through VA facilities, a challenging population to reach given substantial job demands that may require working up to 80 hours a week.

## Methods

The VA is one of the largest integrated health care systems in the U.S., with more than 8 million enrollees and serving more than 5 million veterans annually in urban and rural settings (9). Currently, the VA provides care at 1,321 health care facilities, including 172 Medical Centers and 1,138 outpatient clinics (10). In 2022, approximately 50,000 physician residents received at least part of their medical training in VA (11).

Our population of interest was internal medical residents with a primary care rotation in a VA facility. We used VA's Corporate Data Warehouse to identify all staff with a provider taxonomy of physician resident who were assigned to a primary care provider panel between December 2020 and July 2021. Further details are described in a prior study (12).

Eligible residents were invited to participate in the survey by email. We obtained contact email addresses collected via VA's Talent Management System (TMS). All VA staff, including trainees not paid by VA, provide contact information through this system. We contacted residents using email addresses from TMS because many residents do not regularly access their VA email.

We sent invitations in monthly batches between December 2020 and July 2021. The invitation included an information statement describing the study, a unique link to the web-based survey, an opt-out link, and a statement that participation was voluntary and data collected confidential. We made up to three follow-up attempts. Respondents could skip any item and could discontinue at any time. Per VA regulations, we did not offer financial incentive for participation. Informed consent was obtained from all participants.

### Survey development and measures

**Figure 1:**
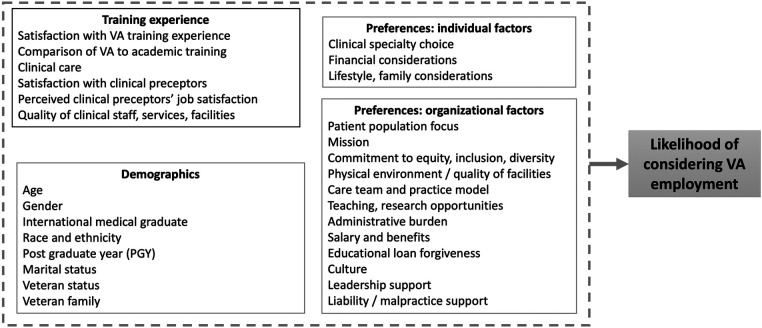
Conceptual model.

We developed a conceptual model to guide our selection of survey measures and to ensure conceptual completeness (Figure 1). We based our model on existing published conceptual models on practice choice, physician satisfaction, and burnout, as well as prior qualitative research highlighting aspects of VA employment that may influence career choice, including the VA mission, culture, care delivery model, and administrative aspects of Federal employment (12–18). The team developed the conceptual model to both build on previous models and highlight aspects of the VA setting. Our conceptual model includes four domains that may influence residents’ choice of VA for post-training employment: VA training experiences, such as overall satisfaction with VA compared to other training environments and perceived quality of clinical care, preceptors, staff, and facilities; individual preferences (e.g., planned specialty, lifestyle and family considerations); and organizational characteristics (e.g., mission, priority population, practice model, benefits, and culture). We also note sociodemographic variables, veteran status, and training year as potential moderators of post-training employment choice.

Where possible, we selected existing measures from VA quality improvement efforts or from the published literature (19). Where *de novo* items were needed, we adapted existing items or used team consensus to develop and refine items. We pilot tested the survey with a convenience sample of three internal medicine residents and incorporated their feedback in the final survey. A copy of the survey is included in Supplementary Material File S1.

Our primary outcome was likelihood of considering future VA employment. We created a *de novo* item with six response options: “How likely would you consider future employment at a VA medical facility?”

VA training experiences included questions measuring trainees’ satisfaction with different aspects of their VA training rotation. To capture VA training experiences, we adapted items from the VA Trainee Satisfaction Survey (20) and the Learners’ Perceptions Survey (19, 21). These instruments have been used internally to assess the educational experiences of VA health profession trainees, and include a 5-point Likert scale with options ranging from “very satisfied” to “very dissatisfied”. Items included satisfaction with VA clinical faculty/preceptors, satisfaction with specific aspects of VA training and clinical environment, comparison of VA experience with academic affiliated training and a global measure of overall satisfaction.

To assess individual preferences for future work conditions, we adapted items from two internal VA survey instruments routinely administered to final year medical residents (22). We also added several constructs from our conceptual model and from previous qualitative work: mission, culture, patient care model, ease of use of the electronic health record system, availability of support staff, fellowship opportunities, leadership opportunities, expected career longevity and patient population. Response options for all preference questions were a 5-point scale: “extremely important” to “not at all important” (12).

Survey questions capturing demographics and Veteran status were adapted from the 2020 Census, National Survey of Veterans, or published literature.

### Analysis

We compared responders and non-responders using the 2-sample t-test for age and the chi-square statistic for sex, census region, month of invitation, and type of email address. We recoded the primary outcome to a dichotomous variable, defining “inclined to work for VA” as endorsement of either “I have already decided to work at VA”; “very likely,” or “likely”. We conducted bivariate analysis, comparing respondent characteristics, preferences, and VA training experiences according to inclination to work for the VA. Training satisfaction and work preferences items were dichotomized as the top 2 of 5 categories defined as satisfied or important. We adjusted for nonresponse bias using inverse probability weights obtained from logistic regression including age, sex, Census region, and type of email address (personal, academic, VA) as predictors.

To conduct multivariable analysis, we used least absolute shrinkage and selection operator (LASSO) regression to identify the strongest predictors from respondent characteristics and measures of satisfaction and importance (23). The LASSO procedure shrinks the coefficient estimates of less important predictors toward zero and are effectively dropped from the model. This process is an automated approach to addressing collinearity in multivariable models. For variables retained by the LASSO procedure, we used logistic regression to assess their association with the dependent variable. We used multiple imputation methods to account for missing data. A nominal p-value of 0.05 was used to determine statistical significance. We used STATA version 16.0 (STATACorp, College Station, TX) for all analyses.

The VA Puget Sound Institutional Review Board approved this study.

## Results

A total of 4,545 residents met inclusion criteria and were invited to complete the survey. We received 268 responses, representing a 5.9% response rate. Active refusal rate was 1.4% (*n* = 65); 242 invitations (5.3%) were returned as undeliverable, and 59 people (1.3%) were determined to be ineligible. Responders were more likely to be female compared to non-responders (51% vs. 43%, *p* = 0.007) but were otherwise similar in age, VA Census region, month of invitation, and type of email address used to complete the survey (Supplementary Material File S2). Complete data was available for 223 (83%) of the sample.

The final analytic dataset included 268 medical residents. This sample was 44% (113 people) female gender; 8% (20 people) Hispanic ethnicity; 25% (60 people) Asian race; 5% Black race (12 people); 1% other race (3 people); and 61% white race (149 people) (Table 1). For marital status, 41% (102 people) reported being single, while 55% (137 people) were married or living with a partner. Two percent (6 people) reported Veteran status; 19% (48 people) reported being from a Veteran family, and 57 people (22%) reported being an international medical school graduate. One hundred and forty-nine people (57%) reported being in their first (81 people) or second (68 people) post-graduate year.

**Table 1:** Population characteristics and post-residency plans.

Variable	Overall (*n* = 268)	Inclined to work for VA^a^ (*n* = 141)	Not inclined to work for VA (*N* = 127)	*P* value
Age (years) mean (SD)	31 (5)	32 (6)	31 (4)	0.72
Gender				0.64
Female	113 (44%)	61 (44%)	52 (44%)	
Male	134 (52%)	73 (54%)	61 (52%)	
Other (trans, gender queer, other, prefer not to answer)^b^	8 (3%)	3 (2%)	5 (0.4%)	
Race				0.006
Hispanic	20 (8%)	17 (13%)	3 (3%)	
Non-Hispanic White	149 (61%)	76 (58%)	73 (65%)	
Non-Hispanic Black	12 (5%)	9 (7%)	3 (3%)	
Non-Hispanic Asian	60 (25%)	30 (23%)	30 (27%)	
Non-Hispanic other	3 (1%)	0 (0%)	3 (3%)	
Marital status				0.65
Never married	102 (41%)	53 (39%)	49 (43%)	
Married, living with partner	137 (55%)	75 (55%)	62 (54%)	
Separated, divorced, or widowed	6 (2%)	5 (4%)	1 (1%)	
Prefer not to answer	5 (2%)	3 (2%)	2 (2%)	
Veteran	6 (2%)	3 (2%)	3 (3%)	0.54
Veteran family	48 (19%)	27 (20%)	21 (18%)	0.79
Post graduate year				0.017
1	81 (31%)	37 (27%)	44 (37%)	
2	68 (26%)	32 (23%)	36 (30%)	
3	71 (28%)	40 (29%)	31 (26%)	
4	20 (8%)	15 (11%)	5 (4%)	
≥ 5	16 (6%)	13 (9%)	3 (3%)	
Training				0.64
U.S. Medical School Graduate	206 (78%)	112 (79%)	94 (77%)	
International Medical School Graduate	57 (22%)	29 (21%)	28 (23%)	
How likely would you consider future employment at a VA medical facility?				<0.0001
I have already decided to work at the VA	19 (7%)	19 (13%)	0 (0%)	
Very Likely	54 (21%)	54 (38%)	0 (0%)	
Likely	68 (26%)	68 (48%)	0 (0%)	
Neutral	48 (18%)	0 (0%)	48 (40%)	
Unlikely	32 (12%)	0 (0%)	32 (27%)	
Very Unlikely	22 (8%)	0 (0%)	22 (18%)	
I have already decided not to work at the VA	11 (4%)	0 (0%)	11 (9%)	
NA	7 (3%)	0 (0%)	7 (6%)	
Which of the following best describes you today?				0.42
I have decided where I will work after residency	73 (28%)	40 (28%)	33 (27%)	
I am close to deciding where I will work after residency	42 (16%)	20 (14%)	22 (18%)	
I am somewhat undecided where I will work after residency	89 (34%)	53 (38%)	36 (30%)	
NA	59 (22%)	28 (20%)	31 (25%)	
What is your intended medical specialty?				
Primary care	64 (24%)	51 (36%)	13 (10%)	<0.0001
Hospital Medicine	90 (34%)	53 (38%)	37 (29%)	0.14
Other	151 (56%)	67 (48%)	84 (66%)	0.002
To what extent are you willing to live in a rural area?				0.29
Very willing	20 (8%)	13 (9%)	7 (6%)	
Willing to live for certain period	83 (32%)	50 (36%)	33 (28%)	
Would rather avoid	115 (45%)	54 (39%)	61 (51%)	
Never	32 (12%)	16 (12%)	16 (13%)	
NA	8 (3%)	5 (4%)	3 (2%)	

^a^ “Inclined to work for VA” Defined as participant endorsement of either “I have already decided to work at VA” or “very likely” or “likely” in response to the survey question “How likely would you consider future employment at a VA medical facility?”. Response options: I have already decided to work at the VA/Very Likely/Likely/Neutral/Unlikely/Very Unlikely/I have already decided not to work at the VA/Undecided.

VA, Veterans Health Administration; U.S., United States; NA, not applicable.

^b^ Categories collapsed to protect privacy.

Fifty four percent (141 people, 54%) reported an inclination to work for VA after residency. Degree of decidedness on plans after residency and willingness to live in a rural area were similar between the inclined and not-inclined groups, as were distributions of demographic characteristics except for race and ethnicity. The group inclined to work for VA was more likely to report Hispanic ethnicity, but less likely to report non-Hispanic White or non-Hispanic Asian race (*p* = 0.006). VA-inclined residents were more likely to declare plans to pursue primary care (36% vs. 10%, *p* < 0.001).

Reported experiences with VA training varied between the inclined and not-inclined groups. Items with the largest differences included overall experience [89% vs. 48% (*p* < 0.001) reporting very satisfied or satisfied]; ability to get patients needed care (87% vs. 51%, *p* < 0.001); quality of clinical staff and services (73% vs. 38%, *p* < 0.001), quality of patient care (94% vs. 61%, *p* < 0.001) and trainee onboarding experience (56% vs. 27%, *p* < 0.001). Ratings of preceptors’ career satisfaction, modeling of work-life balance, patient-orientation, clinical skills, research mentoring, teaching ability, and overall preceptor ratings were all more likely to be highly rated in the VA incliner group (Table 2).

**Table 2:** Satisfaction with VA training experience and preceptors.

	Total (*n* = 268)	Inclined to work for VA^a^ (*n* = 141)	Not inclined to work for VA (*n* = 127)	*P* value
Thinking about the VA facility where you trained/are training, rate your satisfaction				
Overall experience of your VA training (*n* = 261)	68%	89%	48%	<0.0001
Ability to get your patients the care they need (*n* = 263)	69%	87%	51%	<0.0001
Quality of clinical staff and services (*n* = 260)	54%	73%	38%	<0.0001
Quality of care your patients receive (*n* = 263)	78%	94%	61%	<0.0001
Trainee onboarding experience (*n* = 263)	43%	56%	27%	<0.0001
Continuity with patients (*n* = 263)	79%	91%	69%	<0.0001
Quality of non-clinical staff and services (e.g., HR, tech support) (*n* = 263)	46%	57%	38%	0.007
Ownership/personal responsibility for your patients care (*n* = 262)	83%	93%	75%	0.0001
Personal safety (*n* = 262)	84%	92%	75%	0.0004
Physical environment (e.g., clinic rooms, offices, public spaces) (*n* = 263)	45%	54%	38%	0.021
Appreciation of your work by patients (*n* = 262)	84%	92%	76%	0.002
Relationship with patients (*n* = 263)	89%	95%	85%	0.008
Please rate your satisfaction with your clinical faculty/preceptors at the VA facility.				
Seemed happy with career at VA (*n* = 260)	82%	93%	69%	<0.0001
Modeling work-life balance (*n* = 262)	84%	93%	72%	0.0001
Overall satisfaction with your clinical faculty/preceptors (*n* = 260)	88%	97%	78%	<0.0001
Patient-oriented (*n* = 262)	85%	95%	76%	0.0001
Clinical skills (*n* = 263)	84%	92%	76%	0.002
Research mentoring (*n* = 260)	43%	50%	34%	0.022
Teaching ability (*n* = 262)	83%	91%	76%	0.003
Approachability/openness (*n* = 262)	87%	94%	80%	0.004
How would you compare your academic affiliate clinical training experience to the VA clinical training experiences?				
Academic affiliate a lot better	66 (25%)	62 (24%)	10 (7%)	<0.0001
Academic affiliate somewhat better	86 (32%)	84 (32%)	45 (32%)	
Academic affiliate about the same	83 (31%)	81 (31%)	57 (41%)	
Academic affiliate somewhat worse	20 (7.4%)	20 (8%)	18 (13%)	
Academic affiliate a lot worse	2 (0.74%)	2 (1%)	1 (1%)	
Not applicable	12 (4.5%)	12 (5%)	9 (6%)	

^a^ “Inclined to work for VA” Defined as participant endorsement of either “I have already decided to work at VA” or “very likely” or “likely” in response to the survey question “How likely would you consider future employment at a VA medical facility?”. Response options: I have already decided to work at the VA/Very Likely/Likely/Neutral/Unlikely/Very Unlikely/I have already decided not to work at the VA/Undecided.

VA, Veterans Health Administration; HR, human resources; NA, not applicable.

By contrast, individual preferences for working conditions, and family and lifestyle concerns were largely similar between the two groups. Work/life balance, support from leadership and staff, organizational culture, and support from staff and other providers all were rated as important or extremely important by more than 90% of respondents. The importance of compensation and benefits; support from leadership and staff; quality of medical facilities; organizational culture and commitment to equity, inclusion, diversity; geographic location including place to raise a family and partner's career plans; career development potential and fellowship/research opportunities; productivity expectations; patient population served; administrative burden and ease of use of the electronic health record system; and patient care model were all similar in both the VA inclined and VA not-inclined groups. The only factors more highly endorsed as important by residents inclined to work for VA were support for malpractice/liability (85% vs. 69%); work/life balance (97% vs. 90%); expected career longevity (88% vs. 74%) and teaching opportunities (82% vs. 70%) (Table 3).

**Table 3:** Individual preferences considered when making practice decisions.

When you decide where to practice in the future, to what extent are the following important to you? Please select one response for each factor. (1 = Not important at all; 5 = Extremely important)	Total (*n* = 268)	Inclined to work for VA^a^ (*n* = 141)	Not inclined to work for VA (*n* = 127)	*p*-value
Support from leadership/department (*n* = 260)	94%	94%	94%	0.95
Culture of organization (*n* = 262)	94%	92%	94%	0.50
Work/life balance (*n* = 262)	93%	97%	90%	0.010
Availability of support staff (*n* = 261)	92%	90%	90%	0.92
Availability of support from other providers (*n* = 263)	92%	91%	88%	0.59
Ease of use of Electronic Medical Record (EMR) system (*n* = 262)	89%	84%	91%	0.15
Administrative burden (e.g., documentation, clinical reminders, consult process) (*n* = 263)	88%	85%	90%	0.23
Career development potential (*n* = 263)	88%	86%	89%	0.48
Geographic location (*n* = 260)	85%	88%	83%	0.33
Expected career longevity (*n* = 262)	82%	88%	74%	0.012
Number of hours worked (*n* = 263)	82%	85%	79%	0.22
Additional benefits (e.g., retirement, life insurance) (*n* = 263)	82%	85%	78%	0.24
Income/compensation (*n* = 262)	79%	79%	79%	0.99
Teaching opportunities (*n* = 262)	78%	82%	71%	0.042
Patient care model (e.g., integrated health system) (*n* = 262)	78%	81%	73%	0.20
Good place to raise a family (e.g., educational environment) (*n* = 263)	78%	81%	74%	0.26
Support for malpractice/liability (*n* = 263)	77%	85%	69%	0.007
Organization's commitment to equity, diversity, and inclusion (*n* = 263)	76%	79%	72%	0.19
Leadership opportunities (*n* = 263)	71%	71%	70%	0.85
Partner's career and/or preference (*n* = 262)	70%	74%	65%	0.16
Fellowship opportunities (*n* = 263)	65%	60%	68%	0.23
Patient population served	62%	67%	59%	0.24
Mission of organization (*n* = 262)	61%	67%	54%	0.06
Productivity expectations (*n* = 263)	60%	63%	57%	0.41
State-of-the-art medical facilities (*n* = 263)	56%	56%	52%	0.55
Research opportunities (*n* = 263)	52%	55%	47%	0.22
Availability of educational loan forgiveness (262)	45%	48%	42%	0.34

^a^ “Inclined to work for VA” Defined as participant endorsement of either “I have already decided to work at VA”, or “very likely” or “likely” in response to the survey question “How likely would you consider future employment at a VA medical facility?”. Response options: I have already decided to work at the VA/Very Likely/Likely/Neutral/Unlikely/Very Unlikely/I have already decided not to work at the VA/Undecided.

In multivariable analysis, intent to practice primary care was the single most influential variable (OR 4.04, *p* < 0001). Preceptors’ modeling of work-life balance (OR 3.23, *p* = 0.009) and perceptions of quality of clinical staff and services (OR 2.64, *p* = 0.004), ability to get patients the care they need (OR 2.51, *p* = 0.017), and quality of patient care (OR 2.30, *p* = 0.075) were all independent predictors of being in the VA inclined group (Table 4).

**Table 4:** Adjusted results: predictors of self-reported inclination for future VA employment.

	Odds ratio	Std. Err.	*P* > t
Intent to practice primary care	4.04	1.59	<0.001
Preceptors modeling work life balance	3.23	1.44	0.009
Quality of clinical staff and services	2.64	0.89	0.004
Ability to get patients the care they need	2.51	0.97	0.017
Quality of care your patients receive	2.30	1.08	0.075
Availability of educational loan forgiveness	1.44	0.46	0.244
Non-White Race	1.41	0.51	0.343
Support for malpractice liability	1.31	0.49	0.463
Post-Graduate Year (PGY)			
PGY5	3.56	2.80	0.106
PGY4	2.33	1.55	0.203
PGY3	1.85	0.73	0.120
PGY2	1.22	0.48	0.606
PGY1	Ref		

## Discussion

To assess determinants of post-residency inclination to practice at VA, we conducted a cross-sectional, self-administered survey among medical residents receiving residency training at a VA facility. We found that more than half of the sample reported inclination to consider VA for future clinical practice. While individual preferences for work conditions and family/lifestyle factors were remarkably similar for both VA incliners and not-incliners, VA training experiences were markedly more positive in VA incliners. In multivariable analysis, only intent to practice primary care, preceptors’ modeling of work-life balance, perceived quality of clinical staff and services, and ability to get patients the care they need were independent determinants of being in the VA incliner group.

Our study is consistent with existing theoretical models of physician specialty choice that outline training experiences, and inclination toward or away from primary care as important factors in specialty choice (14, 16). Surprisingly, individual preferences for employment were not associated with residents being more or less inclined to work for VA, suggesting that VA may be a feasible option for employment among internal medicine residents with a wide variety of personal preferences.

Previous qualitative research suggests several individual work environment preferences that could influence employment choices such as the decision to work for VA. This includes qualitative research finding identification with the mission of an organization being associated with inclination to work at VA (12). Another study found that among physicians, identification with the institutional culture or values may be an important factor of intent to stay in one's position (17). A qualitative study of factors contributing to primary care physician satisfaction found fulfilling patient-physician relationship and agency in the work environment were key factors in thriving (24). Despite these prior findings, our results indicate that individual work environment preferences were not a statistically significant predictor of inclination to work for VA, after controlling for resident demographics and training experiences.

Conversely, a 2020 systematic review of determinants of burnout in trainee physicians found that workload, concerns about patient care, poor work environment, and poor work-life balance were all predictors of burnout (25). We did not directly measure burnout or other psychological variables, but it is possible that association of poor VA training experience and lack of inclination to work for VA may be related to burnout and warrants further investigation (26, 27).

Our findings suggest that efforts to enhance trainee experiences might both improve medical education and have indirect benefits of highlighting VA as a desirable place to begin a long-term career. One example is the implementation of the Account Provisioning and Deprovisioning System, which centralizes the numerous verification functions required to give trainees access to all the VA systems. Based on information from the VA Medical Informatics Unit (written communication, April 2024, unreferenced), the goal of this system is to have all trainees fully onboarded and ready to work in their clinical rotations on the first day of their training experience, and is tracked using a metric called “Day One Readiness.” Our study adds a unique focus on the importance of specific aspects of physician training experience (e.g., being able to get patients the care they need) as determinants of choice to practice in that same setting by identifying the influential role of such factors during their training rotation.

We note the low response rate as a limitation and cannot rule out the presence of nonresponse bias whereby people who responded had meaningfully different unmeasured characteristics than those who did not respond. Survey response rates have decreased over time, and do not necessarily indicate presence of nonresponse bias (28). Low response rates are not unexpected given the demands of medical residency and the assignment of multiple institutional email addresses during training. However, we tried to anticipate and adjust for potential nonresponse bias. We compared available demographics (age, gender, VA Census region, month of invitation, and type of email address used to complete the survey) to the entire sampling frame, of which only gender was substantially different for responders, and we used sampling weights that adjusted for potential nonresponse bias (29, 30). Further, the rate of active refusal was low (1.4%), suggesting that many people may not have seen the email invitation. As a cross-sectional study we cannot assess changes over time or any causal relationships. Further, we did not assess measures of burnout or other psychological measures of well-being, which may have influenced individual responses about training experiences.

## Conclusions

Our study finds intent to practice primary care and VA residency training experiences are stronger determinants of inclination to consider VA for post-residency practice than individual preferences for work environment or demographic factors. These results provide data-driven insights to help leaders with the strategic recruitment of physicians within and outside of VA. Study results suggest that efforts to maximize the experiences of residency training at the VA may be an avenue for increasing and retaining the primary care workforce serving veterans. Our findings may also inform non-VA systems that serve as training sites by identifying the key role of positive training experiences in effectively recruiting residents into full time positions, particularly primary care practice. Future research could examine longitudinal patterns in career choice, directly tracking practice setting choice, using objective measures in employment data, and exploring negative VA training experiences more deeply.

## Data Availability

The datasets presented in this article are not readily available. Survey data generated by residents are not publicly available because these data contain potentially identifying information, and residents were assured that the information they provided would be publicly available only in aggregate. Questions about the datasets should be directed to VA Puget Sound IRB, VAPugetSoundResearch@VA.GOV.
