# Supplementary material for: Inclination to pursue Veterans Health Administration for primary care practice: survey of medical residents
Source: Front Health Serv. 2024 Jul 18;4:1394072. doi: 10.3389/frhs.2024.1394072 (PMC11291321; doi:10.3389/frhs.2024.1394072)
Supplement: Supplementary file 1 [file Datasheet1.pdf]

Strengthening the VA Physician Workforce

Page 2: Survey

1. Are you a:

- U.S. Medical School Graduate
- International Medical School Graduate

2. What is your Post Graduate Year (PGY)?

3. Which of the following best describes you today? (select one)

- I have decided on where I will work after residency
- I am close to deciding on where I will work after residency
- I am somewhat undecided on where I will work after residency
- I am completely undecided on where I will work after residency

4. Thinking about the VA facility where you trained/are training, rate your satisfaction with the following areas: (If you trained at more than one VA facility, think about your most recent training experience.)

	Very Dissatisfied (1)	2	3	4	Very Satisfied (5)
Trainee onboarding experience	○	○	○	○	○
Physical environment (e.g., clinic rooms, offices, public spaces)	○	○	○	○	○
Quality of clinical staff and services	○	○	○	○	○
Quality of non-clinical staff and services (e.g. HR, tech support)	○	○	○	○	○
Continuity with patients	○	○	○	○	○
Ownership / personal responsibility for your patients' care	○	○	○	○	○
Relationship with patients	○	○	○	○	○
Appreciation of your work by patients	○	○	○	○	○
Quality of care your patients receive	○	○	○	○	○
Ability to get your patients the care they need	○	○	○	○	○
Personal safety	○	○	○	○	○
Overall experience of your VA training	○	○	○	○	○

5. Please rate your satisfaction with your clinical faculty/preceptors at the VA facility in the following areas. Please check one box for each line below.

	Very Dissatisfied (1)	2	3	4	Very Satisfied (5)
Clinical skills	○	○	○	○	○
Teaching ability	○	○	○	○	○
Research mentoring	○	○	○	○	○
Approachability / openness	○	○	○	○	○
Modeling work-life balance	○	○	○	○	○
Seemed happy with career at VA	○	○	○	○	○
Patient-oriented	○	○	○	○	○
Overall satisfaction with your clinical faculty/preceptors	○	○	○	○	○

6. How would you compare your academic affiliate clinical training experience to the VA clinical training experiences?

- ○ Academic affiliate a lot better
- ○ Academic affiliate somewhat better
- ○ Academic affiliate about the same
- ○ Academic affiliate somewhat worse
- ○ Academic affiliate a lot worse
- ○ Not applicable

7. How likely would you consider future employment at a VA medical facility?

- I have already decided to work at the VA
- Very Likely
- Likely
- Neutral
- Unlikely
- Very Unlikely
- I have already decided not to work at the VA
- Undecided

8. When you decide where to practice in the future, to what extent are the following important to you? Please select one response for each factor.

	Not Important At All (1)	(2)	(3)	(4)	Extremely Important (5)
Geographic location	○	○	○	○	○
Partner's career and/or preference	○	○	○	○	○
Good place to raise a family (e.g., educational environment)	○	○	○	○	○
Work/life balance	○	○	○	○	○
Number of hours worked	○	○	○	○	○
Income / compensation	○	○	○	○	○
Additional benefits (e.g., retirement, life insurance)	○	○	○	○	○
Availability of educational loan forgiveness	○	○	○	○	○
	Not Important At All (1)	(2)	(3)	(4)	Extremely Important (5)
Mission of organization	○	○	○	○	○

Culture of organization					
Organization’s commitment to equity, diversity, and inclusion					
Patient care model (e.g., integrated health system)					
Patient population served					
Productivity expectations					
Administrative burden (e.g., documentation, clinical reminders, consult process)					
Ease of use of Electronic Medical Record (EMR) system					
	Not Important At All (1)	(2)	(3)	(4)	Extremely Important (5)
State-of-the-art medical facilities					
Availability of support staff					
Availability of support from other providers					
Support from leadership / department					
Support for malpractice / liability					
Career development potential					
Fellowship opportunities					
Research opportunities					
	Not Important At All (1)	(2)	(3)	(4)	Extremely Important (5)
Teaching opportunities					
Leadership opportunities					
Expected career longevity					
Enter another option (option					
Enter another option (option					
[illegible]					

Enter another option (optiona	○	○	○	○	○
Enter another option (optiona	○	○	○	○	○
Enter another option (optiona	○	○	○	○	○

9. How has differential treatment of yourself or your colleagues based on race, ethnicity, gender, or disability shaped your career choices, if at all?

Note: Your responses will remain confidential and your anonymity will be protected. We will not share any information you provide here. If you would like to report specific instances, contact:

For more information regarding discrimination, contact the Equal Employment Opportunity counselor at 1-888-56-NEW VA (1-888-566-3982).

For more information regarding harassment, contact the Harassment Prevention Program at 1-888-56-NEW VA (1-888-566-3982).

10. How has the COVID-19 pandemic affected your career choices, if at all?

11. To what extent are you willing to live in a rural area?

- Very willing
- Willing to live for certain period of time
- Would rather avoid
- Never
- Other - Please describe

12. How old are you?

13. What is your intended medical specialty? (select all that apply)

- Primary Care (family medicine, internal medicine, pediatrics)
- Hospital Medicine
- Other specialty - Please describe

14. What is your gender identity?

- Female
- Male
- Transgender
- Genderqueer
- Intersex
- Prefer not to answer
- Other - Write In

15. Are you of Hispanic, Latino, or Spanish origin?

- No, not of Hispanic, Latino or Spanish origin
- Yes, Mexican, Mexican American, Chicano
- Yes, Puerto Rican
- Yes, Cuban
- Yes, another Hispanic, Latino or Spanish origin (Salvadoran, Dominican, Colombian, Guatemalan, Spaniard, Ecuadorian, etc.)
- Prefer not to answer

16. What is your race? (select all that apply and write-in specific origin as desired)

- ☐ White
- ☐ Black or African American
- ☐ American Indian or Alaska Native
- ☐ Asian
- ☐ Pacific Islander
- ☐ Some other race
- ☐ Prefer not to answer
- ☐ Other (optional write-in)

17. What is your marital status?

- ○ Single, never married
- ○ Married or living with partner
- ○ Separated or divorced
- ○ Widowed
- ○ Prefer not to answer

18. Are you a military Veteran?

- ○ Yes, on active duty in the past, but not now
- ○ Yes, now on active duty
- ○ No, never on active duty

19. Do you have an immediate family member – a spouse, parent, sibling or child – who has served or is currently serving in the military?

- Yes
- No

20. Which of the following categories best describes the area you spent the most time in when you grew up? (select all that apply)

- Large city
- Midsize city
- Small city
- Town/Village
- Remote area/islands

21. Do you have any comments that you would like to add?
