# Supplementary material for: Inclination to pursue Veterans Health Administration for primary care practice: survey of medical residents
Source: Front Health Serv. 2024 Jul 18;4:1394072. doi: 10.3389/frhs.2024.1394072 (PMC11291321; doi:10.3389/frhs.2024.1394072)
Supplement: Supplementary file 2 [file Datasheet2.docx]

# Supplemental File 2: Table of characteristics of responders and non-responders

| Variable | Entire Sampling Frame  (n=4551) | Responders  (n=268) | Non-Responder  (n=4283) | p-value |
| --- | --- | --- | --- | --- |
| Age (years) | 31 (5)  (n=2981) | 31 (5)  (n=264) | 31 (5)  (n=2641) | 0.019 |
| Women | 1569/3594 (44%) | 138/268 (51%) | 1431/3326 (43%) | 0.007 |
| **Census Region** |  |  |  | 0.27 |
| Midwest | 1038/4444 (24%) | 62/261 (24%) | 1976/4183 (23%) |  |
| Northeast | 639 /4444(14%) | 34/261 (13%) | 605/4183 (14%) |  |
| South | 1645/4444 (37%) | 85/261 (33%) | 1560/4183 (37%) |  |
| West | 1121/4444 (25%) | 80/261 (31%) | 1041/4183 (25%) |  |
| **Month of Invitation** |  |  |  | 0.82 |
| Dec 2020 | 390/4550 (9%) | 21/268 (8%) | 379/4282 (9%) |  |
| Jan 2021 | 3386/4550 (74%) | 209/268 (78%) | 3177/4282 (74%) |  |
| Feb 2021 | 47/4550 (1%) | 3/268 (1%) | 44/4282 (1%) |  |
| April 2021 | 47/4550 (1%) | 2/268 (1%) | 45/4282 (1%) |  |
| June 2021 | 26/4550 (1%) | 1/268 (< 1%) | 25/4282 (1%) |  |
| July 2021 | 644/4550 (14%) | 32/268 (12%) | 612/4282 (14%) |  |
| **Type of Email Address** |  |  |  | 0.06 |
| Personal | 2041/3787 (54%) | 133/230 (58%) | 1908/3557 (54%) |  |
| University | 736/3787 (19%) | 51/230 (22%) | 685/3557 (19%) |  |
| VA | 1010/3787 (27%) | 46/230 (20%) | 964/3557 (27%) |  |
